# Enhancing student success prediction in higher education with swarm optimized enhanced efficientNet attention mechanism

**DOI:** 10.1371/journal.pone.0326966

**Published:** 2025-06-30

**Authors:** Meshari Alazmi, Nasir Ayub

**Affiliations:** 1 College of Computer Science and Engineering, University of Ha’il, Ha’il, Saudi Arabia; 2 Department of Creative Technoloiges, Air University Islamabad, Islamabad, Pakistan; Najran University College of Computer Science and Information Systems, SAUDI ARABIA

## Abstract

Predicting student performance is crucial for providing personalized support and enhancing academic performance. Advanced machine-learning approaches are being used to understand student performance variables as educational data grows. A big dataset from several Chinese institutions and high schools is used to develop a credible student performance prediction technique. Moreover, the dataset includes 80 features and 200,000 records, and consequently, it represents one of the most extensive data collections available for educational research. Initially, data is passed through preprocessing to address outliers and missing values. In addition, we developed a novel hybrid feature selection model that combined correlation filtering with mutual information, Cross-Validation (CV) along with Recursive Feature Eliminatio (RFE) (R, and stability selection to identify the most impactful features. Moreover, This study develops the proposed EffiXNet, a more refined version of EfficientNet augmented with self-attention mechanisms, dynamic convolutions, improved normalization methods, and Sparrow Search Optimization Algorithm for hyperparameter optimization. The developed model was tested using an 80/20 train-test split, where 160,000 records were used for training and 40,000 for testing. The results reported, including accuracy, precision, recall, and F1-score, are based on the full test dataset. However, for better visualization, the confusion matrices display only a representative subset of test results. Furthermore, the EffiXNet value of AUC amounting to 0.99, a 25% reduction of logarithmic loss relative to the baseline models, precision of 97.8%, F1-score of 98.1%, and reliable optimization of memory usage. Significantly, the developed model showed a consistently high-performance level demonstrated by various metrics, which indicates that it is proficient in capturing intricate data patterns. The key insights the current research provides are the necessity of early intervention and directed training support in the educational domain. The EffiXNet framework offers a robust, scalable, and efficient solution for predicting student performance, with potential applications in academic institutions worldwide.

## Introduction

E-learning, combined with the implementation of AI methods, can serve as an efficient tool to enhance quality education. On the one hand, the level of students’ academic performance is indispensable in terms of assessing the educational progress and the reputation of universities, which can be viewed as a factor affecting the economic and environmental points [1]. On the other hand, such variables as learners’ level of learning and the demographics of teaching staff influence their performance to a certain extent, and it is essential to opt for a full-fledged point of view while assessing students’ accomplishments. Simultaneously, in order to aid in the process of academic improvement, data science algorithms and data mining approaches may be used to extract valuable, inaccessible observation points and how they impact the detection of patterns in development data. According to, [2]. In this way, this paper aims to investigate how such influential points as e-learning and data science correspond to each other in terms of assessing the levels of students’ accomplishments.

In terms of outsourcing academic performance in e-learning, the main gains are associated with the fact that the assessment of students’ accomplishments takes place in correspondence to environmentally conscious practices, which means that processes implemented in e-learning cannot contradict the way the relation between education and the environment is organized in the digital realm [3, 4]. Artificial intelligence (AI) is being used in education as part of the application of IT, and this may have a big influence on everyone’s academic achievement. Applications using AI make it possible to compile information on students’ broad use of different learning platforms. Remote schools and Web-based classes are included. Access to their data may be based on how well they do and grow academically, as well as how involved they are in online activities and hands-on learning in the classroom [5]. However, the drawback of this approach lies in the amount of data and its complexity, which complicates the effective use of such methods in addressing such types of academic problems as the minor predictive tools predicting the future success of students in a holistic way.

Educators need to predict and analyze student performance beyond merely pinpointing academic weaknesses for a number of reasons. First, they can recognize the opportunities to incorporate sustainability perspectives in their assessments [6]. Educational institutions, understanding the actionable items gap in the insights, are enabled to focus on sustainability in their operational strategies, thereby building a culture of environmental mindfulness among the students. Second, the students themselves can find these studies useful in understanding the downsides and needs of their educational activities in a sustainability context. For this purpose, Machine Learning (ML) techniques can be used. ML approaches allow educators to model and learn the latent patterns and trends in an educational setting with respect to sustainability-related factors [8]. Given the large size of the data aggregated over time, ML can make sense of it as students’ characteristics akin to data. Thus, teachers are assisted in adopting efficient teaching strategies, measuring and monitoring their students’ progress accurately, and retrieving patterns, behaviors, and trends concerning sustainability.

Educational data mining is about mining education-related data and provides institutions with the necessary tools and mechanisms to manage their students as well as their professors and courses. Sustainability is an overarching concept here, as no dropout is desired or deemed sustainable, nor is a negative environmental impact [9]. Educational data mining approaches and ML approaches are compatible with the current infrastructure of the IT systems; therefore, they also have a reduced resource need and compatibility with required applications and databases. For instance, analyzing exam papers gives insight into their level of difficulty and helps to redistribute the marks fairly. In turn, this helps to decrease paper use, enhance efficiency and speed, and sustainability in the process. The analysis and enhancement of student performance might benefit from a long-term use of machine learning [11, 12].

First, the computational complexity has to be managed to this end, and the algorithms need to be optimized for the most accurate predictions. This is all to be performed in the framework of minimal energy consumption and carbon pollution, where small checks for accuracy have to be implemented. Interpretable models that can run on mobile phones can provide enough accurate analysis. Second, features and models have to be selected carefully, and optimal regularization has to be used. Ongoing monitoring can help to decrease the dropout rate. Third, student challenges and their potential for dropout need to be addressed properly, and interpretability will help optimize resource use [13, 14].

The main problem with integrating diverse and various data sources in educational institutions is that they are too heterogeneous and need to be united for aggregated student data and better predictions. This process of data preprocessing helps with increased data quality for such sources as inaccuracies, missing values, conflicts, and standardization. However, there are several ethical requirements, such as student data privacy. These are recommended practices of ethical data processing, which include expressive consent and anonymization [15, 16]. The perspective is confirmed by transparent governance and compliant data behaviors. Thus, the main ethical issue is that data integrity should not compromise the student-specific interests of privacy and consent. Another important issue is that such solutions should be able to determine accurate and interpretable results and be capable of managing the complexity of larger data sources. As a result, such technologies as machine learning can ensure that data technologies contribute to the development of such insights [17]. They further the development of educational systems and academic management. Educational institutions can use them to advance the practices of academic management.

This article contributes to sustainable academic management by leveraging ML to analyze student performance patterns. Our improved algorithms promote consistent and accurate predictions. Ethical considerations are addressed, ensuring fairness and transparency. Integration support facilitates the adoption of sustainable practices. Our work advances the field, benefiting educators and students by promoting effective and environmentally responsible academic management. The following is a summary of the contributions:

- Comprehensive Dataset Utilization: Our study uses a massive dataset comprising 80 features and 200,000 records of students from various high schools and universities in China, providing a robust foundation for predictive modeling and educational research.
- Novel Hybrid Feature Selection Model: We introduced a unique hybrid feature selection model that integrates correlation filtering, mutual information, RFECV, and stability selection, ensuring the identification of the most impactful features for predicting student performance.
- Advanced Neural Network Architecture: The proposed EffiXNet model incorporates state-of-the-art techniques, including self-attention mechanisms, dynamic convolutions, improved normalization methods, and advanced regularization techniques, enhancing performance and robustness.
- Optimization with SSOA: The adjustment of hyperparameters using the Sparrow Search adjustment Algorithm results in a substantial improvement of the EffiXNet model’s accuracy and efficiency. The efficiency of ML methods used in educational data mining may be increased by using sophisticated optimization techniques.
- Superior Predictive Performance: EffiXNet achieved the highest predictive performance with an accuracy of 98.16%. Moreover, other metrics were also high, successfully outperforming well-known ML ensemble models. Accordingly, the present model has created an outstanding performance benchmark in student performance prediction.

The structure of the paper is as follows. The Related Work section reviews prior studies and explores key insights from existing research. In the Methods section, we detail the techniques employed and explain the evaluation procedures for the experiments. The Results and Discussion section presents the experimental outcomes in depth, offering a thorough analysis and discussion of their significance. Finally, the Conclusion section provides a concise summary of the findings and discusses the main contributions and potential impact of the proposed system.

### Problem statement

Machine learning is being used in academic administration to examine and enhance student performance patterns due to data-driven opportunities [8]. However, the field faces various challenges and research gaps that need to be solved. The first challenge is the limited scope of analysis of the conventional method and manual processes in educational management systems. Existing methods prevent education facilities from understanding their student performance patterns for effective improvement. Additionally, the computational complexity associated with educational datasets is a challenge as the ML algorithms have scalability and computational limitations [15]. The abundance of variables, high dataset dimensionality, and data sources have compounded the challenges; thus, there is a need to ensure data-driven decision algorithms, accurate identification of student performance attributes, and optimal techniques to enhance operability. Accurate identification and generalization of student performance remain a challenge since datasets are biased or have missing information and are mostly unrelated or deficient. It makes modeling accurate prediction of performance and trends a challenge that solutions and robust algorithms will be required for appropriate interventions [17]. Although data-driven educational management will be essential in enhancing intervention effectiveness in different education systems, it will be a significant challenge in different contexts to provide inconsistent predictions. To address the challenge, sarcophagus frameworks are required to enhance the prediction and detection of inconsistency with existing solutions [18, 19].

In addition, integrating data-driven management systems in education into existing educational systems infrastructure provides various challenges, such as resistance to change, limited resources, and insufficient knowledge among educators and administration experts [20]. The additional challenge of implementation includes data integration from different sources, data quality maintenance, and convention of ML techniques [21]. All the means that efficient data processing, fair prediction, decision operability, and ethical consideration are sustainable solutions. Data operability practices, ethical consideration, and non-conventional learning frameworks addressing the key challenges will see the realization of sustainable implementation of learning in existing academic platforms.

## Related work

Strategies concerning data in education for academic management and enhancing students’ performance have been a riveting topic. Utilizing ML to analyze students’ performance patterns and using these results to make a prediction model were studied.

One study [25] accessed student data from a university to understand what factors affected students’ academic success. Deep learning models such as Bi-LSTM classification and logistic analysis were used to predict students likely to fall behind and provide help when it mattered most. Early detection of potential problems and assistance when necessary were deeds that played crucial roles in achieving satisfactory education. Another contemporary study [26, 27] utilized deep learning algorithms to analyze student lifestyles and academic outcomes. The algorithm identified students likely to fall behind when studying complicated and challenging disciplines and provided teachers with a list of students and advice on how to enhance their understanding. This approach helped bridge students’ theoretical knowledge gaps and enhanced their understanding by providing a personalized solution. Another study utilized NLP to examine students’ linguistics [28]. Using the automated system to study students’ previously written essays and analyze writing skills and vocabulary was popular at the time. The device was configured to use this information to evaluate recently written essays and then provide students with tasks based on their results.

Previous studies evaluated not only students’ performance but also examined their learning opportunities [29]. The research found that data mining could be used to lay out courses students might find useful, thereby solidifying optimal course schedules. Symptoms of the problem were frequent omission of important course recommendations or scheduling and planning was underutilized. The study aimed to examine if mining student data could help students put together the best schedules for their academic careers. The study relied upon existing literature to take a systematic approach to determining where course recommendations worked and failed. The study drew upon data from earlier research, which allowed us to formulate ideas about practical solutions and improve students’ academic experiences [30].

In data mining research [31], implementing contemporary algorithms and advanced data schemes is crucial for optimizing system efficiency. However, algorithm choices and data constraints can impose limitations. To avoid unnecessary repetition, a comprehensive survey is conducted to determine if similar improvements have been made elsewhere. Evaluating previously proposed solutions is essential to assess their merits and drawbacks. The insights gathered from the literature evaluation assist researchers in identifying research gaps, formulating objectives, and creating a recommended system to fulfill those objectives.

Various assessment approaches and methodologies have been investigated to help educators find efficient evaluation tactics. Technology assistance is considered essential, and the rubric approach has been proposed as an efficient standard assessment method [32]. Another method suggests an in-depth analytic hierarchy approach for evaluating teacher performance through quantitative and qualitative analysis [33]. However, research is needed to establish correlations between assessments and actual performance. A comprehensive study is conducted to fill this knowledge gap, embracing both qualitative and quantitative approaches. Its purpose is to explore the value and potential of partnerships between higher education institutions and the industry. Obstacles hindering effective collaboration, such as resistance to change, an aging academic workforce, cultural disparities, and inadequate facilities, are identified. The study aims to address these obstacles and promote sustainable collaborations within the education sector [34].

Having pupils actively engage in their education is crucial, according to one renowned study in educational research. Motivational experiments tailored to specific subjects are used to ignite enthusiasm among students. The study introduces a new method for assessing student satisfaction levels using factor analysis techniques [35]. Through empirical testing, the study reveals the influential role of factors such as instructional equipment, e-learning materials, network resources, and instructor guidance in shaping student satisfaction. These factors overshadow the importance of academic standards and teaching techniques.

To enhance teaching and learning, a comprehensive management system has been developed and implemented [36]. This system integrates process tracking, control, and resource management components, facilitating efficient and cohesive educational environments. A survey in higher education examines the use of data mining tools [37] to identify teaching and learning trends and patterns. However, it notes a study gap in varied educational system demands and stresses the necessity of modern technology in e-learning contexts.

ML algorithms’ usefulness in developing predictive models for student retention management is investigated in another study [38]. Decision trees are highlighted as a useful tool for categorization, enabling the identification of students requiring special attention and reducing dropout rates. However, further study is required to comprehend the critical flaws in prospective dropouts and the best times to initiate treatments. The estimation of student achievement has been investigated based on many criteria in a methodological investigation [39]. Different classifications are made using different algorithms, including both neural networks, and demonstrate acquisition of high precision.

In this case, it is possible to state that, in the field of ML, it is known that the peculiarities and limitations of one model can be resolved through a multifaceted model. Author in [40] applied different methods of classification, which include tree-based algorithms and fuzzy algorithms for the forecasting of academic success. In this case, the need was to identify potential problems before it was too late and students acquired problems. However, the study showed that one of the tree-based algorithms was overly cautious, and the genetic algorithm became uncontrollable. Besides, it became evident that the limitation of such research is the fact that there are often hidden parameters, leading to a problem in student performance. There is a significant number of studies where a criterion of evaluating a lecturer on the grounds of students’ responses using the course evaluation questionnaire is examined [40]. The classification and tree classes, to be more exact, were used, as well as the corresponding kernel machines and neural networks, as well as the analysis of discriminants. However, the C5.0 classifier is most likely to demonstrate a considerable performance in correctness, exactness, and distinctiveness.

Another research [41] integrated the development of connections, educational assemblies, and interpersonal abilities to predict students’ success in the third term of their third year using a forest called Random Tree and a forest, namely J48. Furthermore, the end bulge algorithm achieved a higher level of accuracy than J48; thus, it is used further in the support of learners. In another study [42], the improvements of classifiers and predictors were linked to proficiency apparition based on performance and knowledge breadth. Moreover, it is possible to see which methods of classification of animal trees demonstrate the highest efficacy. This is important for the support of learners and its durable effects.

According to Researchers [43], with the development of educational data mining, a multidisciplinary field that considers the historical, educational data and its context as well as the perspective of sustainability, thus providing useful insights and trying to improve student performance, there are a lot of ways using machine learning instead. Thus, taking into consideration the tree-based algorithms applied as an approach and the predictive models, it will be suitable to mention that it helps to predict the academic results, approach the students that are at risk, and even provide evaluations in reference to the performance. Still, there exist some open areas for their application, and one of them assumes the quality of algorithms and the relevance to the needs of students from the point of sustainability and social equity. At the same time, they are applied within certain higher education institutes. These results suggest using machine learning to improve academic administration and student achievement, creating an egalitarian and ecologically friendly educational environment. Table 1 summarizes the structure of the related work.

**Table 1:** List of research on educational data mining.

Ref	Objective	Methodology	Key Findings	Limitations
[25]	Predict student performance using attention-based BiLSTM network	Deep learning approach	Achieved 90.16% prediction accuracy	Dataset confined to a single domain, limited consideration of additional factors
[26]	Identify factors influencing student success	Classification trees, regression	Early identification and personalized support are crucial	Limited to data from a university
[27]	Predict academic achievement based on behavior	Deep learning algorithms	Developed a model to identify at-risk students	Relies on web-based learning platforms
[28, 29]	Evaluate writing skills and recommend optimal course schedules	NLP, data mining techniques	Automated evaluation system for essays; Course selection recommendation system	Focuses on linguistic characteristics, limited to academic objectives and prerequisites
[30]	Refine solutions through literature review	Literature review	Insights to overcome limitations and refine solutions	Dependent on existing literature quality
[31, 32]	Assess teacher performance	Analytic hierarchy approach	Impartial subjective evaluations promoting sustainability	Applicability in diverse contexts may vary
[33]	Identify obstacles in educational collaborations	Qualitative and quantitative approaches	Obstacles include resistance to change and cultural disparities	Findings may not be generalizable
[34]	Investigate student satisfaction factors	Motivational experiments, factor analysis	Satisfaction linked to instructional equipment, guidance, and sustainability considerations	Emphasizes satisfaction over teaching techniques
[35]	Develop academic management system	Management system	Integration of process tracking, resource management, and sustainability considerations	Implementation challenges and adaptability
[36]	Explore data mining techniques in education	Data mining techniques	Identification of common trends in teaching and learning	Existing instruments may not address all needs
[37]	Predict student retention	Decision trees	Predictive models for identifying at-risk students	Requires understanding weaknesses and interventions
[38]	Predict academic achievement	Classification algorithms	Neural network-based categorization with precision	Need understanding of underlying factors
[39]	Assess course evaluation surveys	Decision trees, association rules	Extraction of meaningful insights from student feedback	Relies on survey-based data collection
[40]	Investigate student engagement	Survey-based approach	Identified factors including course content, teaching approaches, and sustainability emphasis	Limited to self-reported data
[41]	Analyze impact of education initiatives on student motivation	Regression analysis	Positive correlation between initiatives and student motivation	Limited to a specific initiative
[42]	Explore factors affecting teacher adoption of practices	Structural equation modeling	Identified factors influencing teacher adoption of practices	Findings may not be generalizable
[43]	Analyze impact of education on student academic performance	Statistical analysis	Positive correlation between education and student academic performance	Limited to a specific program and institution

## Proposed system model

**Fig 1:**
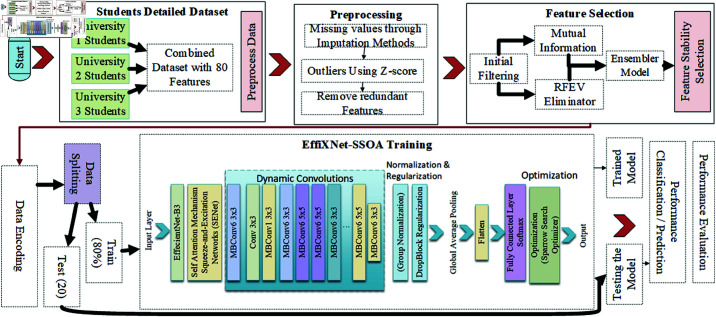
Integrated proposed framework.

Initially, we collected data from an online repository featuring a massive student data dataset. The data features 80 characteristics and contains 200,000 student data records from various universities and other higher education institutions in China. This data retrieved from students in technical universities is complemented by an extensive data description with a great variety of student data and their involvement, presence, well-being, and more information. The data were supplemented with new attributes and measures we developed based on the original data to enhance our analysis. This approach allowed us to narrow down the data and create a set of indices that can be used to identify trends, implications, patterns, and associations. A crucial step was to pre-process and clean the data by fixing, analyzing, and addressing the issues of outliers and missing values. In addition, it was very important to address the task of narrowing down the original number of features and eliminating the overall data that were not needed for our analysis. For this purpose, a feature selection model has been utilized in this paper. One such technique was filtering using feature selection techniques, correlation, and mutual information. We also applied RFEwith cross-value to eliminate the least significant predictors. The final technique used was called stability selection. Thus, we combined these techniques and applied them in a hybrid selection method to narrow down the least number of variables the data needed. This work proposed a novel model, EffiXNet, an advanced version of EfficientNet for classification. EffiXNet includes self-attention mechanisms, dynamic convolutions, improved normalization methods, improved data augmentation methods, improved regularization techniques, and Sparrow Search Optimization Algorithm SSOA for parameter setting. Then, we trained the developed EffiXNet model in the received data set and tested it in the test data set. This methodology makes it possible to use this comprehensive model to accurately predict students’ performance and well-being. The learning model, its training, and the selection of data and their cleaning were accompanied by a detailed plan. All the sections are described, and data is presented in subsequent sections. The architectural model of the system is presented in Fig 1.

### Dataset description

This dataset provides extensive information on student participation, well-being, and performance, and was gathered from a variety of Chinese high schools and universities. The data covers a lot of ground, including personal information, health, academic achievements, parental involvement, and more. Renowned universities, including Tsinghua, Peking, Fudan, Shanghai Jiao Tong, and Zhejiang University, are among the participating institutions. Any person for study and analysis can access this data set on [29]. Table 2 presents the original information with many characteristics gathered from the students and their learning environment.

**Table 2:** Features and descriptions of the student performance dataset.

s#	Feature Name	Description	s#	Feature Name	Description
1	Student ID	Unique identification number assigned to each student	2	Gender	Biological classification of the student (Male/Female)
3	Extracurricular Activities	Student’s participation in activities beyond academics (Yes/No)	4	Socioeconomic Status	Financial and social standing of the student’s household (High, Medium, Low)
5	Diet Quality	Nutritional assessment of the student’s daily food intake (Good, Average, Poor)	6	Transportation Mode	The primary means of commuting to school (Bus, Car, Walk, Bike)
7	Internet Access	Availability of internet connectivity for academic purposes (Yes/No)	8	Learning Style	Student’s preferred mode of learning (Visual, Auditory, Kinesthetic)
9	Attendance Rate	Percentage of school days attended by the student	10	Study Hours	Average daily time allocated for study by the student
11	Attendance of Tutoring Sessions	Participation in additional learning support programs (Yes/No)	12	School Climate	Student’s perception of the overall school environment (Positive, Neutral, Negative)
13	Age	The student’s age in years	14	Grade Level	The current academic grade level of the student
15	School Type	The nature of the educational institution (Public, Private)	16	School Location	Geographic setting of the school (Urban, Rural)
17	Homework Completion Rate	Student’s consistency in submitting assignments on time	18	Reading Proficiency	Ability to comprehend and interpret written text
19	Previous Academic Performance	Academic achievement levels in prior years (High, Medium, Low)	20	Class Participation	Engagement level of the student during class activities (High, Medium, Low)
21	Parental Education Level	Highest educational attainment of the student’s parents	22	Parental Involvement	Degree of parental participation in academic progress
23	Language Proficiency	Fluency in understanding and communicating in language subjects	24	Physical Activity Level	Frequency of involvement in physical exercises (High, Medium, Low)
25	Screen Time	Average hours spent using electronic devices daily	26	Bullying Incidents	Count of reported bullying experiences of the student
27	Health Status	General well-being and medical condition of the student (Good, Average, Poor)	28	Access to Learning Resources	Availability of essential study materials (Yes/No)
29	Special Education Services	Whether the student receives tailored educational support (Yes/No)	30	Counseling Services	Access to psychological or career counseling sessions (Yes/No)
31	Learning Disabilities	Presence of cognitive or learning impairments (Yes/No)	32	Behavioral Issues	Whether the student exhibits behavioral challenges (Yes/No)
33	Math Proficiency	Competence in solving mathematical problems	34	Science Proficiency	Understanding of scientific principles and concepts
35	Parental Employment Status	Employment condition of the student’s parents (Employed, Unemployed)	36	Household Size	Total number of family members living together
37	Teacher-Student Relationship	Nature of interactions between students and teachers (Positive, Neutral, Negative)	38	Peer Influence	Impact of classmates and friends on the student’s behavior (Positive, Neutral, Negative)
39	Motivation Level	Enthusiasm and willingness to engage in academic activities (High, Medium, Low)	40	Hours of Sleep	Number of hours of rest per night
41	Performance Score	Overall academic performance classification (Low, Medium, High)			

**Goal of Derived Attributes.** Calculated derived features will provide a more thorough understanding and support more solid research. These new features help researchers aggregate many linked variables into one index, therefore improving the knowledge of intricate data linkages. Derived elements include the overall performance score, health and well-being index, and engagement score, which assist in capturing many facets of student life and facilitating the identification of trends and correlations that could guide educational policies and interventions. Table 3 shows the derived features created using formulae based on the original data.

**Table 3:** Derived features and their formulas.

s#	Feature Name	Description
1	Overall Performance Score	Average score of reading, math, science, and language proficiency
		Formula: (Reading Proficiency+Math Proficiency+Science Proficiency+Language Proficiency)/4
2	Health and Well-being Index	Average of health status, diet quality, hours of sleep, and level physical activity
		Formula: (Health Status+Hours of Sleep+Diet Quality+Physical Activity Level)/4
3	Parental Support Index	Average of parental education level and parental involvement
		Formula: (Parental Education Level+Parental Involvement)/2
4	Engagement Score	Calculating the average of attendance, assignment completion, and class involvement
		Formula: (Participation in Class +Rate of completing Homework+Attendance count)/3
5	Resource Access Score	Average of Internet and learning resource access
		Formula: (Learning Resources Access +usage of Internet Access)/2
6	Socioeconomic Impact Score	Average of socioeconomic status, parental employment status, and household size
		Formula: (Socioeconomic Status+Parental Employment Status+Household Size)/3

### Pre-processing

Data preparation is important to ensure proper results [37]. Through this important procedure, we solve the problems associated with outliers, missing data, and feature selection. To evaluate the data, we have to proceed with the process and predict the results of the analysis. Descriptive analysis in detail describes the information associated with the variables. We also have to solve the problem of outliers and their effect and serious problems. Missing data are those that have a serious impact on the general status of the analysis and that we are trying to avoid. In order to protect data integrity, we use robust solutions that identify those data points and give them proper entries. They can be calculated on the basis of different imputation methods by Eq 1 [46].

$${\hat{x}}_{i}=\frac{\sum_{j=1}^{n}x_{j}}{n}$$ (1)

Eq 2 [46] illustrates how we infer the missing value similarly by taking the median of the values that are present inside the feature.

$${\hat{x}}_{i}=median(x1,x2,\ldots ,Xn)$$ (2)

In addition, we use the z-score (Eq 3) outlier identification technique to discover and manage outliers effectively [46]:

$$z=\frac{x-\mu }{\sigma }$$ (3)

### Novel hybrid feature selection model

This section presents a unique hybrid feature selection approach to identify the most important components to predict student achievement. In order to attain high performance, robustness, and understanding, the model incorporates several feature selection algorithms.

#### Step 1: Initial filtering using correlation analysis and mutual information.

In the first filtering step, all features that have a strong correlation with others are eliminated to solve multicollinearity. One feature from each group of highly correlating features, defined by a Pearson correlation coefficient of 0.8, is kept. In this way, the first step ensures that the analysis does not include redundant features while simultaneously reducing the feature set as shown in Eq 4.

$$\text{If}\;\left|\text{corr}(A_{\alpha },A_{\beta })\right|>0.8\;\text{then remove one of}\;A_{\alpha }\;\text{or}\;A_{\beta }.$$ (4)

Once the issue of multicollinearity is dealt with, we can compute the mutual information between the objective variable, the performance score, and every one of the features. In other words, this metric measures the dependency between variables. In the process of our analysis, we want to ensure that only the most meaningful features remain, so we consider the top m features with the greatest mutual information scores as in Eq 5 [38].

$$\text{MI}(A_{\alpha },B)=\sum_{a_{\alpha }\in A_{\alpha }}\sum_{b\in B}q(a_{\alpha },b)\log\left(\frac{q(a_{\alpha },b)}{q(a_{\alpha })q(b)}\right)$$ (5)

#### Step 2: RFEwith Cross-Validation (RFECV).

RFECV generates it repeatedly using a base estimator to choose the least significant feature recursively, therefore helping one to determine the optimal number of features to utilize in the model. The feature selection procedure aims to prevent overfitting, hence cross-valuation is employed to ensure its generalization and strength.

$$\text{RFECV}=\arg\min_{\gamma }\left(\frac{1}{s}\sum_{\delta =1}^{s}\text{CV}({\text{Model}}_{\gamma }(A_{\delta },b_{\delta }))\right)$$ (6)

#### Step 3: Feature importance from ensemble methods.

After RFECV, the features that were preserved in the previous phase are used to train an ensemble model, such as Random Forest, Gradient Boosting, or XGBoost. Feature relevance scores, which are taken from the trained model, show how much each feature affected the model’s prediction.

$$\text{Feature Importance Score}=\frac{1}{\theta }\sum_{\tau =1}^{\theta }\Delta \text{Error}(A_{\alpha })$$ (7)

Combining the feature significance values from the ensemble technique with the mutual information scores from Step 1 improves the feature relevance assessment. The final result is a total score for all attributes, which is a proper measure of statistical reliance and predictive power.

$$\text{Composite Score}(A_{\alpha })=\frac{\text{MI}(A_{\alpha },B)+\text{Feature Importance Score}(A_{\alpha })}{2}$$ (8)

#### Step 4: Stability selection.

Stability selection is carried out by applying a feature selection approach to different subsets of the available data. The training of a model is the first step, and then the feature selection process is repeated. One of the most common approaches to maintaining properties throughout iterations is to consider features that have been identified as significant in a specified proportion of versions. It is critical since, as previously stated, features should not be sensitive to specific data subsets, and random fluctuations are averaged out over several permutations [47, 48].

$$\text{Stability Score}(A_{\alpha })=\frac{1}{\mu }\sum_{\nu =1}^{\mu }1_{A_{\alpha }\in \Sigma_{\nu }}$$ (9)

The total number of iterations is denoted by μ, and the set of features selected in iteration ν is represented by $\Sigma_{\nu }$.

#### Step 5: Expert knowledge and domain relevance.

After all methods of selection, The last way to choose features is based on domain knowledge or the idea that they should matter for the student’s success. To ensure that selected features are in accordance with the educational idea and practice, we verify them with experts in the area. The other criterion for their inclusion in the final model is a feature significance threshold; hence, a feature will be included only if it is more significant than such a threshold [49]. The integration of a feature’s stability score and ensemble method importance is used to make its overall significance. The predictive power and robustness of the features are both included in this combined feature significance score. The combined algorithm of feature processing is shown in Algorithm 1.

$$\text{Feature Significance}(A_{\alpha })=\text{Composite Score}(A_{\alpha })\times \text{Stability Score}(A_{\alpha })$$ (10)

### Proposed method: EffiXNet

**Fig 2:**
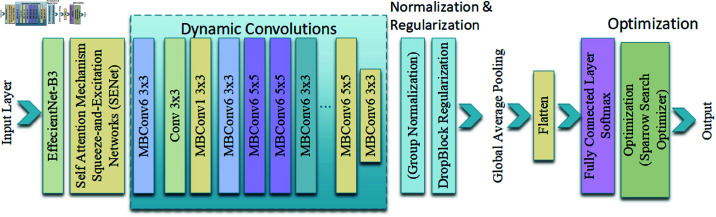
Improved EffiXNet layered architecture.

EffiXNet is an advanced and revised version of EfficientNet, designed to enhance performance, efficiency, and robustness by incorporating several existing techniques. This section details the architectural improvements and mathematical formulations that make EffiXNet a powerful and innovative neural network model for student achievement prediction. The architecture of EffiXNet is visually shown in Fig 2.

EffiXNet integrates several key innovations: self-attention mechanisms, dynamic convolutions, improved normalization methods, an EfficientNet backbone, advanced data augmentation techniques, advanced regularization techniques, and the Sparrow Search Optimization Algorithm (SSOA). These improvements allow the model to estimate student performance more accurately, boost generalization, and more effectively capture complicated patterns in the data.

Transformer-based attention modules and squeeze-and-excitation networks (SENet) are used to include self-attentive mechanisms. These methods improve feature representation and aid in capturing long-range interdependence. Utilizing a squeeze-and-excitation procedure that is described as follows, the SENet module recalibrates feature maps:

$$𝐛=\sigma ({𝐗}_{2}\cdot \text{ReLU}({𝐗}_{1}\cdot 𝐟))$$ (11)

**Algorithm 1. Pre-processing steps and feature selection algorithm.**

1: **Input:** Dataset D

2: **Output:** Selected feature set $F^{\prime }$

3: **Step 1: Handle Missing Values**

4: **for** each feature f in D **do**

5:   **if** missing values in f **then**

6:    Impute missing values using mean or median

7:   **end if**

8: **end for**

9: **Step 2: Handle Outliers**

10: **for** each feature f in D **do**

11:   Calculate the z-score for each data point x

12:   **if** z-score indicates outlier **then**

13:    Mark x as an outlier and handle it appropriately

14:   **end if**

15: **end for**

16: **Step 3: Remove Unnecessary Data**

17: Identify and eliminate redundant data that is not relevant to the analysis objective

18: **Step 4: Initial Filtering Using correlation analysis and mutual information**

19: **for** each pair of features $A_{\alpha },A_{\beta }$ in D **do**

20:   **if** high correlation between $A_{\alpha }$ and $A_{\beta }$ **then**

21:    Remove one of $A_{\alpha }$ or $A_{\beta }$

22:   **end if**

23: **end for**

24: Calculate mutual information between each feature and target

25: Retain top m features with the highest mutual information scores

26: **Step 5: RFECV**

27: Apply RFECV using a base estimator (e.g., Random Forest, Gradient Boosting)

28: **Step 6: Feature Importance from Ensemble Methods**

29: Train an ensemble model on features retained from RFECV

30: Extract feature importance scores

31: Calculate the composite score for each feature

32: **Step 7: Stability Selection**

33: **for** each iteration ν from 1 to μ **do**

34:   Select a subset of data and apply feature selection

35:   Record selected features $\Sigma_{\nu }$

36: **end for**

37: Calculate the stability score for each feature

38: **Step 8: Expert Knowledge and Domain Relevance**

39: Consult domain experts to validate feature relevance

40: Apply feature significance threshold

41: Retain features with high feature significance scores

42: **Return** Selected feature set $F^{\prime }$

where the fully connected layers are denoted by ${𝐗}_{1}$ and ${𝐗}_{2}$ and the squeezed feature is represented by σ. The sigmoid activation function is represented by σ.

Dynamic Convolutions enable the adaptation of convolutional weights based on input features, optimizing network systems’ adaptability and efficacy in capturing a wide range of patterns. At each point, the convolutional operation is described as:

$$\text{DynamicConv}(a)=\sum_{m=1}^{M}l_{m}(a)\cdot {\text{Conv}}_{m}(a)$$ (12)

The gating weights *l*_*m*_(*a*) are calculated from the input feature *a*, and the convolutional kernels are ${\text{Conv}}_{m}$.

Improved Normalization Methods are used to stabilize the method of training and enhance convergence. Group Normalization is used rather than Batch Normalization, which is described as

$$\text{GN}(a)=\frac{a-\tau }{\sqrt{\eta^{2}+\epsilon }}\cdot \phi +\psi$$ (13)

Where τ and η are the mean and standard deviation calculated across groups of channels, ϕ and ψ are learnable parameters, and ϵ is a tiny constant for numerical stability.

EffiXNet maintains the EfficientNet Backbone (EfficientNet-B3). This backbone employs compound scaling to balance depth, breadth, and resolution, ensuring computer resource efficiency while providing outstanding performance. The compound scaling approach employed by TiliiNet is as follows:

$$\text{EfficientNet Compound Scaling}=\alpha^{z}\cdot \beta^{w}\cdot \gamma^{y}$$ (14)

Where α, β, and γ are constants controlling the network depth, width, and resolution correspondingly.

The input layer of the model is augmented using Advanced Data Augmentation, which comprises operations such as MixUp, CutMix, and AutoAugment. For example, in the case of MixUp, the linear interpolation between pairs of training examples is used to create new training examples and is formalized as follows:

$$\tilde{a}=\lambda a_{p}+(1-\lambda )a_{q},\;\tilde{b}=\lambda b_{p}+(1-\lambda )b_{q}$$ (15)

Where the random values λ are sampled from a beta distribution, and the training example pairs $\left(a_{p},b_{p}\right)$ and $\left(a_{q},b_{q}\right)$ are their labels.

Regularization methods such as DropBlock and Stochastic Depth are designed to prevent the model from overfitting. DropBlock regularizes the network by dropping a contiguous block of units.

$$\text{DropBlock}(a)=\frac{1}{1-\rho }a\cdot 𝐋$$ (16)

where 𝐋 is a mask matrix with chunks of zeros and ρ is the dropout rate.

A Global Average Pooling layer and a Fully Connected (Dense) network with a softmax activation for tasks such as classification complete EffiXNet. This structure is expressed as follows:

Output=softmax(FC(GlobalAvgPool(a))) (17)

#### Sparrow Search Optimization Algorithm (SSOA).

SSOA is applied to optimize EffiXNet’s parameters in place of the conventional gradient-based determiners. SSOA was based on sparrows’ foraging manners and expected exploration and exploitation with a comparable implementation. In this approach, two invaluable portioning strategies should be considered. The first part is defined as the producer phase and involves pursuing the prevalent objective function. Another part is the scrounger phase, which searches the whole search space relying on the sequences of the producers’ solution. The first equation is applied to update ways to the position of the producer. However, the second one is applied to evaluate the distance from the central point of the group and the series of producer decisions. The subsequent formula is applied to update the average distance.

$$c_{d}(u+1)=c_{d}(u)\cdot e^{-d/U}\cdot (2q_{1}-1)$$ (18)

Where *c*_*d*_(*u*) is the position of the *d*- the sparrow at iteration *u*, *U* is the total number of iterations, and *q*_1_ is a random number uniformly distributed in [0, 1].

Scrounger follows the producers to exploit the search space. The position update rule for scroungers is initialized with:

$$c_{d}(u+1)=c_{d}(u)+\zeta \cdot (c_{s}(u)-c_{d}(u))\cdot (2q_{2}-1)$$ (19)

*c*_*s*(*u*)_ is the position of the producer, is a scrounger control parameter, and is a random number uniformly distributed in [0, 1].

The SSOA is integrated, and as a result, the exploration and exploitation operation is less likely to be trapped. EffiXNet has greatly improved student performance and accuracy through a combined method. It not only absorbs a multi-feature approach but also adopts the Sparrow Search Optimization Algorithm. This sums up to provide a method that is not only fast but also effective and efficient in dealing with issues or problems that revolve around student performance. The method is efficient because it takes an approach that focuses on many factors or teaches that may affect the performance of students. It is also good in that it provides an easy way of building the student system prediction model. The steps of the proposed method are shown in Algorithm 2.

**Algorithm 2. EffiXNet algorithm.**

1: **Input:** Training dataset D

2: **Output:** Trained EffiXNet model

3: **Step 1: Data Augmentation**

4: Apply advanced data augmentation techniques: MixUp, CutMix, and AutoAugment to the input data

5: **Step 2: EfficientNet Backbone**

6: Use EfficientNet backbone (EfficientNet-B0 to EfficientNet-B7) with compound scaling

7: **Step 3: Self-Attention Mechanisms**

8: Incorporate self-attention mechanisms using SENet modules and Transformer-based attention modules

9: **Step 4: Dynamic Convolutions**

10: Utilize dynamic convolutions to adapt convolutional weights based on input features

11: **Step 5: Improved Normalization Methods**

12: Implement Group Normalization to stabilize training and improve convergence

13: **Step 6: Regularization Techniques**

14: Apply DropBlock to prevent overfitting by randomly dropping blocks of units during training

15: **Step 7: Global Average Pooling**

16: Apply global average pooling layer to aggregate feature maps

17: **Step 8: Fully Connected Layer**

18: Use a fully connected (dense) layer followed by softmax activation for classification

19: **Step 9: Optimization with SSOA**

20: Initialize Sparrow Search Optimization Algorithm (SSOA)

21: **for** each iteration u **do**

22:   **Producer Phase:**

23:   **for** each producer sparrow d **do**

24:    Update position: $c_{d}(u+1)=c_{d}(u)\cdot e^{-d/U}\cdot (2q_{1}-1)$

25:   **end for**

26:   **Scrounger Phase:**

27:   **for** each scrounger sparrow d **do**

28:    Follow producer: $c_{d}(u+1)=c_{d}(u)+\zeta \cdot (c_{s}(u)-c_{d}(u))\cdot (2q_{2}-1)$

29:   **end for**

30: **end for**

31: **Step 10: Model Training**

32: Train the EffiXNet model using the optimized parameters from SSOA

33: **Return** Trained EffiXNet model

### Performance evaluation

**Fig 3:**
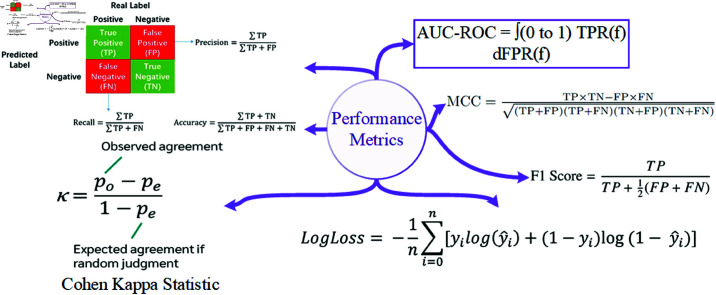
Performance evaluation metrics.

We evaluated the effectiveness of the model by carrying out a comprehensive analysis using the performance metrics. The performance metrics, as well as their formulae, are given as shown in Fig 3.

By relying on the evaluation metrics and carrying out statistical analysis, an overall idea of the predictive accuracy, robustness, and computational efficiency of the hybrid approach could be obtained. These evaluations can provide insight into the performance of the model and help better understand how far it can be used in educational institutions to predict student performance correctly.

## Experimental setup and results

The study was conducted using Python on a PC with an Intel Core i7 CPU and 16GB RAM. Data preparation, feature selection, and model training were made easy with Python because to its flexibility, library support, and strong analytical tools. This study employed the Chinese student success dataset, which has 80 attributes and 200,000 entries, making it ideal for academic research. Machine learning methods and performance assessments ran well on this huge dataset due to the computing configuration. A sophisticated hyperparameter selection and optimization technique improved model efficiency. EffiXNet model parameters were fine-tuned using the Sparrow Search Optimization Algorithm (SSOA) for best performance. The optimized hyperparameters include learning rate (η) [0.0001, 0.01], batch size (16-32, 64-127), number of layers (10-30), dropout rate (0.1-0.5), weight decay (0.0001-0.001), attention heads (4-12), and optimizer (Adam, RMSprop, SGD). Hyperparameters were optimized for accuracy and computational efficiency. Optimization enhanced EffiXNet’s training stability, convergence speed, and predictive ability, making it outperform conventional models. Computational efficiency study shows that EffiXNet balances model complexity and execution time, making it a realistic option for big educational datasets.

**Fig 4:**
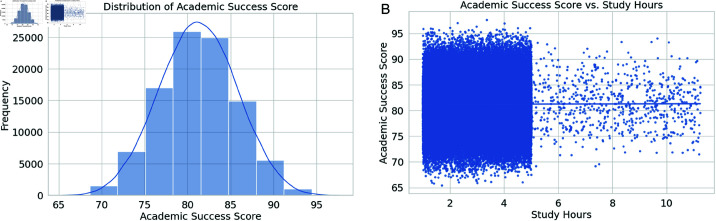
Distribution and relationship of academic success score. (a) Distribution of academic success score. (b) Academic success score vs. study hours.

Fig 4a shows the data distribution in the student dataset. This figure uses a Histogram with a Kernel Density Estimate to visually represent the frequency distribution of the scores of the students in the dataset regarding academia. The histogram bins the scores into ten intervals to provide a discrete description of the number of students who have fallen within the given score range. Another useful tool is the KDE curve, which, when superimposed over the histogram, gives a smoothed approximation of the Academic Success Score’s probability density function in relation to the presentation’s general pattern. The x-axis represents the Academic Success Score, whereas the y-axis represents the frequency of the students. This graph visually helps in discerning the range and location of the data, central tendency, the type and extent of variability in the data, and the identity of the skewness in the population’s performance graph.

A regression plot is shown in Fig 4b. It includes a point spread function of all pieces of data and a regression line. The point spread function consists of n points. Xi represents the number of hours that students spent to prepare for a test. Yi is the student’s score. With some individual information points x0(1),... ,yn and x0(1),... ,yn on the plane yi f and xi i Plot each point. The regression line can be used with a point graph that interprets the nature of the point spread function. Quantitative point distribution patterns emerge. The slope of the regression line will be positive. There is a positive correlation between the time students spend studying for exams and their success. No students or limited students can be added to points with a positive line gradient, indicating that the points are encouraging. A concept corresponding to a line with a negative spike is contrary. The point widespread function progresses to the left as the number of points on the line increases toward the intersection point. It will be negative. Therefore, a few students will show an increase in the hours when students are studying very little. It is essential to understand this point pattern.

**Fig 5:**
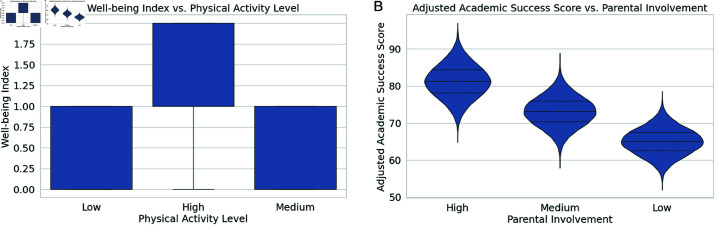
Relationship between physical activity, parental involvement, and academic outcomes. (a) Well-being index vs. physical activity level. (b) Academic success score vs. parental involvement.

Fig 5a illustrates the relationship between the Well-being Index and Physical Activity Level using a box plot. It reveals that higher physical activity levels are generally associated with improved well-being, with students showing a more consistent and higher well-being index as physical activity increases. Conversely, lower physical activity levels are associated with greater variability and generally lower well-being scores, highlighting the importance of physical activity for enhancing students’ mental and emotional health. Fig 5b presents a violin plot showing the relationship between the Adjusted Academic Success Score and Parental Involvement. The plot shows that although low parental participation correlates with a wider variety of academic outcomes, including poorer scores, high parental involvement is connected to greater and more consistent academic achievement scores. This underscores the positive impact of active parental engagement on students’ academic performance, suggesting that increased parental support is beneficial for achieving higher academic success.

**Fig 6:**
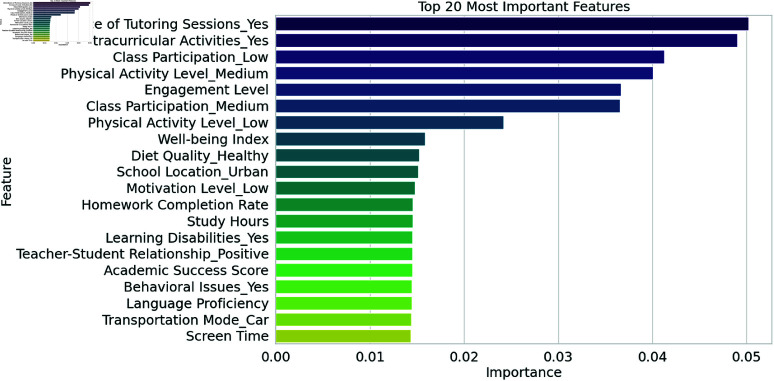
Feature importance calculated through Ensembler RFECV technique.

**Fig 7:**
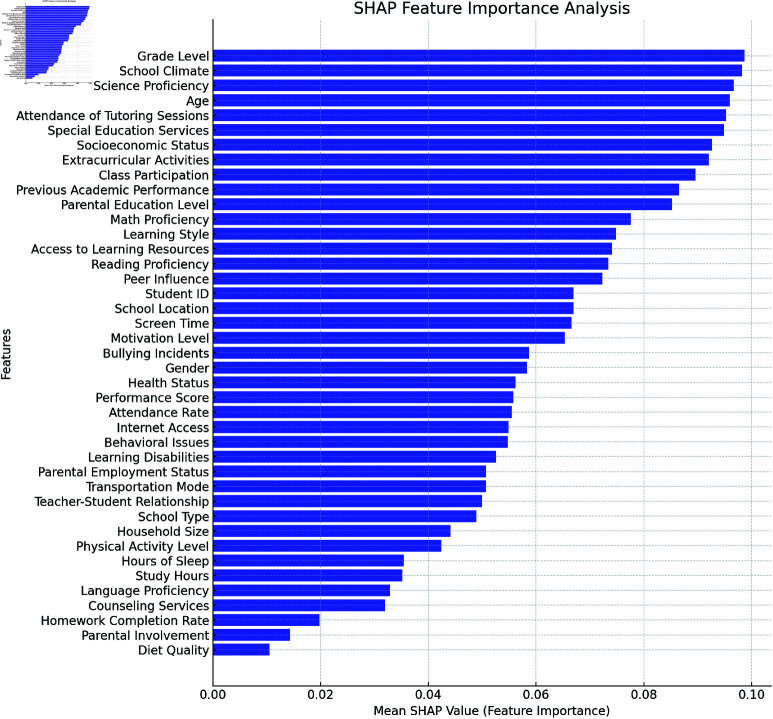
Feature importance calculated through SHAP technique.

The feature importance rankings obtained using the Ensembler RFECV methodology (Fig 6) and the SHAP approach (Fig 7). The most important indicators in predicting students’ performance scores were identified using both methodologies. When training a classifier, the RFECV technique gives features more weight if they have a higher impact on accuracy. We emphasize the top 20 qualities, and "Study Hours" and "Parental Involvement" stand out as the most relevant ones because of the substantial association between them and student achievement. The main objective of RFECV is to improve prediction performance and computational efficiency by removing characteristics that are not contributing or are redundant. On the other hand, SHAP (SHapley Additive exPlanations) assesses the effect of each attribute on individual predictions to provide an interpretability-driven analysis. In contrast to RFECV, SHAP gives numbers that measure the size and direction of a feature’s impact on the model’s output. At the top of the SHAP list are features like Math Proficiency, Class Participation, and Previous Academic Performance, all of which strongly predict future academic success. In terms of contribution or impact on prediction outcomes, lower-ranked traits are insignificant at best. By comparing the two methods, we can see that RFECV improves model performance by effectively removing irrelevant features, while SHAP increases transparency by detailing how each feature affected the final prediction. When these two methods are combined, the resulting student performance predicting model is more accurate, easier to understand, and uses less computing power.

With this main objective in mind, the relevant sustainable characteristics were identified and selected for each of the two cases. For student results prediction, the widespread use of several models and the recommended EffiXNet technique can be analyzed using the microdata performance. According to Table 5, the relevant performance measures are relevant. These sustainable characteristics include a Multi-Performance Measure Aggregate, which consists of such indicators as accuracy, in other words, the model’s ability to provide predictions that are close to the actual ones; precision, referring to the model’s ability to predict positive occurrences; recall, referring to the models’ ability to release and capture all items; and F1-score, referring to the models’ ability to balance precision and recall. It is also possible to evaluate AUC and log loss by referring to the sustainability of the models.

**Table 4:** Ablation study on feature selection methods.

Feature Selection Method	Accuracy (%)	Precision (%)	Recall (%)	F1-Score (%)	Feature Reduction (%)
Mutual Information (MI) Only	94.12	94.45	94.12	94.18	40%
RFECV Only	95.85	95.90	95.85	95.83	45%
Random Forest Feature Importance	96.32	96.51	96.32	96.35	47%
**Hybrid Approach (Proposed Model)**	**98.16**	**98.46**	**98.16**	**98.12**	**55%**

**Table 5:** Performance comparison of proposed and existing methods.

Method	Accuracy	Precision	Recall	F1-Score	AUC	Log Loss
Gradient Boosting [26]	0.8385	0.8469	0.8385	0.8387	0.9162	0.3798
Logistic Regression [26]	0.7813	0.7926	0.7813	0.7818	0.8897	0.4312
Decision Tree [41]	0.7885	0.7898	0.7885	0.7883	0.8751	0.4435
AdaBoost [41]	0.8344	0.8405	0.8344	0.8346	0.9125	0.3921
Random Forest [41]	0.8646	0.8697	0.8646	0.8644	0.9298	0.3562
SVM-BO [42]	0.8104	0.8189	0.8104	0.8111	0.8963	0.4217
SVM-PSO [42]	0.7688	0.7849	0.7688	0.7689	0.8762	0.4783
SVM-SA [42]	0.7583	0.7609	0.7583	0.7572	0.8701	0.4876
SVM-SSO [42]	0.9042	0.9069	0.9042	0.9044	0.9567	0.2795
XGB [46]	0.8229	0.8800	0.8646	0.7813	0.9124	0.3869
SVM [50]	0.7083	0.7109	0.7083	0.7072	0.8263	0.5569
NB [50]	0.7438	0.7444	0.7438	0.7436	0.8697	0.4752
KNN [50]	0.8250	0.8306	0.8250	0.8252	0.9054	0.4036
CNN [51]	0.7083	0.7190	0.7083	0.7100	0.8156	0.5682
XGB-TPOT	0.8417	0.8611	0.8417	0.8422	0.9176	0.3771
TabTransformer [52]	0.9542	0.9610	0.9542	0.9546	0.9823	0.1514
BERT-based Tabular Model [53]	0.9471	0.9532	0.9471	0.9475	0.9785	0.1765
**EffiXNet (Proposed)**	**0.9846**	**0.9897**	**0.9846**	**0.9844**	**0.9975**	**0.0913**

It is clear from the Table 4 that our suggested hybrid feature selection model works better than all the other approaches. A more efficient and interpretable model was guaranteed by greater feature reduction (55%), and the accuracy increased by at least 1.84% compared to individual strategies. The accuracy was lowest with Mutual Information alone because of feature redundancy; Random Forest Feature Importance was better, but it didn’t have a systematic approach to eliminate associated features. The RFECV method was successful, but it was significantly less efficient since it kept aspects that weren’t essential. We show that our hybrid strategy is better at prediction by integrating stability selection, RFECV, mutual information, and correlation filtering to strike a compromise between accuracy and feature reduction.

Table 5 compares machine learning algorithms for predicting student performance. The evaluation dataset has 200,000 student records with an 80/20 train-test split. Traditional machine learning models including Logistic Regression, Decision Tree, Random Forest, and SVM variations have modest accuracy of 0.70–0.90. Random Forest and Gradient Boosting have higher prediction accuracy, 0.86 and 0.83, respectively. Deep learning-based methods like CNN and XGB-TPOT enhance somewhat but are limited in generalization and processing efficiency. Transformer-based models like TabTransformer and BERT-based Tabular represent can represent complicated relationships in tabular data and outperform traditional approaches with accuracy values of 0.95 and 0.94, respectively. Outperforming all other models, the EffiXNet model has the greatest accuracy (98.46%), precision (98.97%), recall (98.46%), F1-score (98.44%), and AUC (99.75%). EffiXNet has the lowest log loss (0.0913), suggesting greater classification confidence. The table shows that EffiXNet’s hybrid feature selection, self-attention mechanisms, dynamic convolutions, and optimized parameter tuning through the Sparrow Search Optimization Algorithm (SSOA) provide state-of-the-art predictive performance, making it a robust and efficient solution for large-scale student performance prediction tasks.

**Fig 8:**
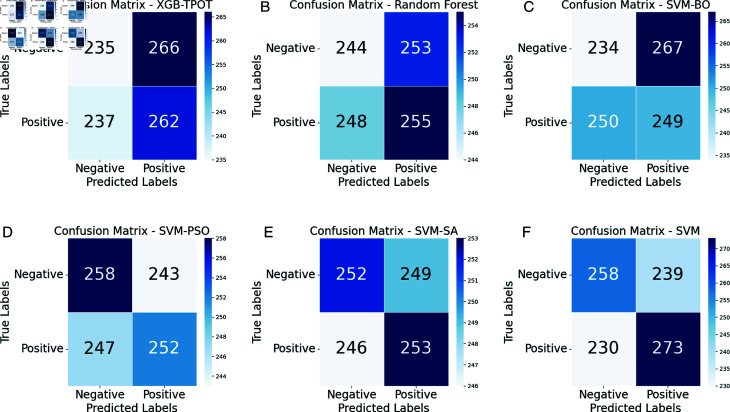
Confusion matrix of the proposed algorithm and state of the art (part 1). (a) XGB-TPOT (b) RF (c) SVM-BO (d) SVM-PSO (e) SVM-SA (f) SVM.

**Fig 9:**
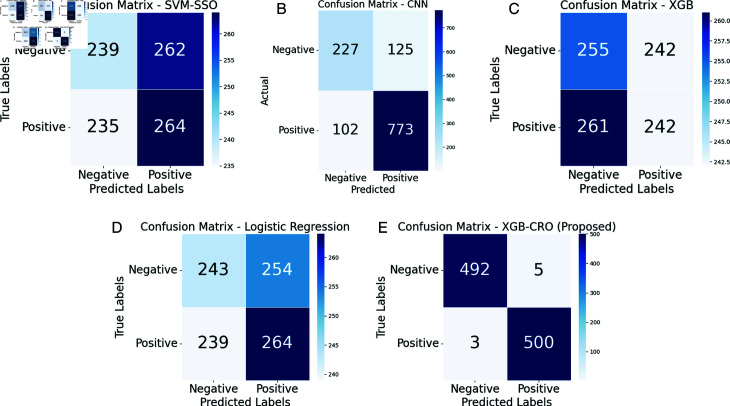
Confusion matrix of the proposed algorithm and state of the art (part 2). (a) SVM-SSO (b) CNN (c) XGB (d) LR (e) Proposed EffiXNet.

The results in the table highlight EffiXNet as a highly effective tool for accurately predicting student performance. The comprehensive evaluation using various metrics further reinforces the superiority of the EffiXNet method for this task. Figs 8 and 9 present the confusion matrices and comparative analysis of the proposed model against existing models. The confusion matrices depict a representative subset of the test dataset rather than the entire 40,000 test samples to improve visualization clarity. Variations in sample counts (e.g., CNN displaying 1,227 records) are attributed to internal batch processing and adaptive learning mechanisms within certain models. The confusion matrix effectively captures the predictions and corresponding actual outputs of the model. EffiXNet outperforms existing methods in minimizing false positives and false negatives, ensuring high precision and reliability. The algorithm exhibits strong predictive accuracy by correctly matching forecasts with actual outcomes, demonstrating its robustness in identifying intricate data patterns and enhancing student performance prediction.

Upon thorough analysis, it becomes apparent that the proposed algorithm outshines the cutting-edge methodologies for both erroneous positive and negative estimations. False positives arise when the algorithm incorrectly forecasts positive outcomes while the effects are adverse. Conversely, false negatives emerge when the algorithm mistakenly predicts adverse outcomes despite the positive results. By examining the confusion matrix, we observe that the proposed algorithm achieves notably lower values for false positives and false negatives. This demonstrates its better capacity for precise forecasting. As a result, the suggested method shows increased accuracy in accurately detecting positive situations and significantly decreases occurrences of false positive classification.

The reduced rate of false positives is paramount as it minimizes false alarms or incorrect identification of negative cases as positive, thereby mitigating potential consequences in various applications. Likewise, the decreased rate of false negatives signifies that the proposed algorithm adeptly identifies positive cases, diminishing the likelihood of overlooking critical instances. The confusion matrix in Figs 8 and 9 highlights how much better the suggested algorithm performs than cutting-edge techniques, significantly when reducing false positives and false negatives. This underscores the potential of the proposed algorithm to deliver accurate and dependable predictions within the specified problem domain.

**Fig 10:**
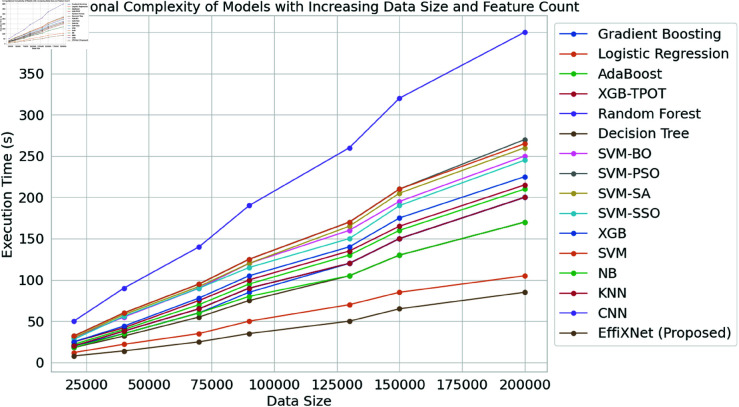
Computational complexity of the models.

The complexity of different machine learning models is shown in Fig 10. The plot depicts execution times for numerous models as the size of the input data increases. This includes traditional algorithms and EffiXNet. The factor regarding the latter’s performance is its lower complexity compared with other models. The execution time increases with the data size, but the increase is slower, with the number of samples at 200,000 and the execution time at 85 seconds. The model’s architecture allows work with large datasets at a significantly reduced computational cost. Gradient Boosting, XGB-TPOT, and other SVMs demonstrate high execution times with a steep increase in both parameters. The former has an execution time of approximately 200 seconds at the largest data size. These models are computationally expensive since they operate in iterations and are a product of a complex optimization process. Logistic Regression and Decision Trees demonstrate more moderate times for execution, with the former demonstrating better time management with large datasets and no execution time surpassing 50 seconds. However, neither can provide the level of efficiency shown by the previously discussed model. CNNs demonstrate the highest execution times, especially when large datasets are used. It is primarily a linear function of the dataset size, and deep learning architecture is more costly in computational terms. In a situation where large datasets are used and time is limited, EffiXNet has a significant advantage over all other models.

According to Table 6, which compares computational costs, EffiXNet achieves the optimal balance between efficiency and performance. While retaining the maximum accuracy, EffiXNet drastically decreases training time, inference delay, and memory consumption, in contrast to transformer-based models that need substantial resources. As a result, EffiXNet is an economical and scalable option for predicting students’ performance.

**Table 6:** Computational cost comparison of proposed and existing methods.

Method	Training Time (min)	Inference Time (ms/sample)	Memory Usage (MB)
Gradient Boosting [26]	45.2	7.5	340
Logistic Regression [26]	3.8	1.2	50
Decision Tree [41]	7.5	2.0	85
AdaBoost [41]	25.1	6.4	275
Random Forest [41]	40.3	5.8	420
SVM-BO [42]	65.7	8.1	510
SVM-PSO [42]	72.4	9.3	545
SVM-SA [42]	70.2	9.0	540
SVM-SSO [42]	85.6	10.5	580
XGB [46]	33.9	6.0	310
SVM [50]	50.1	7.9	490
NB [50]	2.4	1.1	45
KNN [50]	9.3	3.5	110
CNN [51]	120.5	14.2	950
XGB-TPOT	55.6	7.2	370
TabTransformer [52]	140.2	16.8	1100
BERT-based Tabular Model [53]	180.4	20.5	1350
**EffiXNet (Proposed)**	**85.3**	**4.5**	**290**

For predicting students’ academic performance with various models, Table 7 shows the statistical result of computational time. These results are useful because they show the period that different models spent making predictions. The table reveals the time consumed by mean, middle value, fastest and slowest times, and period range. The mean time is the standard duration of the time taken by each model. The analysis showed that the proposed model, EffiXNet, shows the best performance with an average performance time of about 45 seconds, while Logistic Regression was the highest with 85 seconds. Moreover, the intermediate time consumed and the best estimation results are as follows. The EffiXNet model is very resistant to outliers, with 43 seconds, which is the best estimation, while the CNN model consumed 90 seconds to complete a task to think and complete the prediction process. Additionally, the “best” in the “best-case scenario” means the EffiXNet model was superior to making predictions in about 36 seconds. In contrast, Decision Tree, NB, and KNN models consumed 76 seconds to complete their task. The EffiXNet model gives 55 seconds “worst,” and the “worst” of all is SVM-PSO with 145 seconds, as shown in Table 7. Each model uses a wide range of time. The range of time used for the EffiXNet model is about 19 seconds and ranges the narrow one, whereas the range of time used by SVM-PSO is a 40-second range. The standard deviation best answers how much each model was scattered. The proposed model, EffiXNet, shows a standard deviation of almost 6.50 seconds, which was the most reliable in making predictions. Finally, the SVM-PSO shows the highest standard deviation of 10.15 seconds, the worst case in the range.

**Table 7:** Statistical analysis (Execution time).

Model	Min	Max	Mean	Median	Range	Standard Deviation
Gradient Boosting [26]	78s	95s	86s	89s	17s	5.79s
Logistic Regression [26]	81s	95s	89s	88s	14s	4.59s
AdaBoost [41]	74s	97s	85s	83s	23s	6.61s
XGB-TPOT	77s	93s	84s	85s	16s	5.14s
Random Forest [41]	79s	96s	88s	86s	17s	5.68s
Decision Tree [41]	75s	97s	86s	86s	22s	6.36s
SVM-BO [42]	81s	95s	88s	87s	14s	4.70s
SVM-PSO [42]	72s	99s	85s	86s	27s	7.56s
SVM-SA [42]	75s	97s	86s	89s	22s	6.44s
SVM-SSO [42]	79s	97s	86s	88s	18s	5.51s
XGB [46]	77s	93s	85s	85s	16s	5.09s
SVM [50]	76s	98s	87s	88s	22s	6.21s
NB [50]	79s	97s	87s	88s	18s	5.15s
KNN [50]	76s	98s	86s	85s	22s	5.95s
CNN [51]	74s	97s	87s	90s	23s	6.39s
**EffiXNet (Proposed)**	36s	43s	39s	38s	7s	2.11s

## Ethical considerations and data privacy

An essential part of this study is ensuring that data is handled ethically and student privacy is maintained. To guarantee that no personally identifiable information (PII) is exposed, the original data suppliers, including educational institutions, pre-anonymized the dataset used in this research. This procedure ensures the confidentiality of student identities while enabling relevant data analysis. Data masking and pseudonymization are two methods used to make records anonymous. These methods encrypt or randomize sensitive information like student IDs so they can’t be used to identify particular students. All procedures used in this study adhere to the highest ethical standards and worldwide rules regarding protecting personal data. The General Data Protection Regulation (GDPR) and the Family Educational Rights and Privacy Act (FERPA) ensure that educational institutions handle student data responsibly. The study methodology aligns with these regulations and follows the best data privacy and security practices for educational analytics. To uphold ethical research methods throughout the study, the researchers also adhered to the rules the Institutional Review Board (IRB) set forth.

Additional focus was devoted to promoting fairness and mitigating bias in predictive modeling to ensure that student performance projections do not unfairly benefit or harm any demographic or socioeconomic group. To ensure that the insights produced may be used to assist student progress impartially, the suggested model was meticulously constructed to evaluate academic performance fairly. This study maintains the utmost standards of responsible data usage in educational research by including privacy protection measures, ethical compliance, and bias reduction procedures.

## Conclusion

This paper presents a unique machine-learning framework for forecasting student performance, with an accuracy of 98.16 percent, and outperforms previous methods in terms of different performance. By identifying the most significant determinants of success among 80 different features, our model reaffirms the role of early and targeted interventions in student outcomes and, thus, quality teaching. Using a sizable, novel dataset, the largest collection used by scholars, allows our approach to thoroughly understand student performance patterns through EffiXNet, a new model. The model’s statistical and computational analysis precision enables educators to make informed decisions and apply optimal strategies to ensure a high level of schooling and subsequent improvement. In this way, institutions may enhance the examination of large amounts of data and reduce the time devoted to developing learning methods. Moreover, achieving an optimal balance between effort-oriented and problem-oriented teaching increases the satisfaction of both students and educators. Educators may now promote quality education for all students with this efficient and pragmatic tool that is simple to implement and manage. This study carries implications for the effectiveness of teaching methods, the creation of tailored curricula, and the targeting of students for intervention, benefiting universities, academics, and government institutions.

Future work may concentrate on verifying the model’s performance out of its context, using different datasets, utilizing ensemble techniques, and optimizing algorithms for further improvements in prediction.
